# RAC-tagging: Recombineering And Cas9-assisted targeting for protein tagging and conditional analyses

**DOI:** 10.1038/srep25529

**Published:** 2016-05-24

**Authors:** Oliver Baker, Ashish Gupta, Mandy Obst, Youming Zhang, Konstantinos Anastassiadis, Jun Fu, A. Francis Stewart

**Affiliations:** 1 Stem Cell Engineering, Biotechnology Center, Technische Universität Dresden, BioInnovationsZentrum, Tatzberg 47, Dresden 01307, Germany; 2 Genomics, Biotechnology Center, Technische Universität Dresden, BioInnovationsZentrum, Tatzberg 47, Dresden 01307, Germany; 3 Shandong University–Helmholtz Joint Institute of Biotechnology, State Key Laboratory of Microbial Technology, Shandong University, Shanda Nanlu 27, 250100 Jinan, People’s Republic of China

## Abstract

A fluent method for gene targeting to establish protein tagged and ligand inducible conditional loss-of-function alleles is described. We couple new recombineering applications for one-step cloning of gRNA oligonucleotides and rapid generation of short-arm (~1 kb) targeting constructs with the power of Cas9-assisted targeting to establish protein tagged alleles in embryonic stem cells at high efficiency. RAC (Recombineering And Cas9)-tagging with Venus, BirM, APEX2 and the auxin degron is facilitated by a recombineering-ready plasmid series that permits the reuse of gene-specific reagents to insert different tags. Here we focus on protein tagging with the auxin degron because it is a ligand-regulated loss-of-function strategy that is rapid and reversible. Furthermore it includes the additional challenge of biallelic targeting. Despite high frequencies of monoallelic RAC-targeting, we found that simultaneous biallelic targeting benefits from long-arm (>4 kb) targeting constructs. Consequently an updated recombineering pipeline for fluent generation of long arm targeting constructs is also presented.

Gene targeting utilizing endogenous homologous recombination has been central to the emergence of the repertoire of genetic technologies including conditional mutagenesis, ligand-inducible loss-of-function and endogenous protein tagging^1^. However these technologies have been largely restricted to the few model systems that support efficient gene targeting, notably yeast and mouse.

Nuclease-assisted mutagenesis, pioneered by zinc-finger/Fok1 nucleases^2^ and now exemplified by the RNA-guided CRISPR-Cas9 nuclease^3^,^4^, is fundamentally changing genetic engineering. Nuclease-assisted mutagenesis, in particular CRISPR/Cas9, unlocks the advantages of directed mutagenesis for many genomes^5^,^6^,^7^. It also presents new potential for improvements to existing genetic technologies such as gene targeting to establish protein-tagged or conditional alleles. In contrast to the extraordinarily rapid progress with Cas9 nuclease-assisted mutagenesis in many applications, the application of Cas9 to gene targeting has been less straightforward. In contrast to nuclease-assisted mutagenesis, nuclease-assisted targeting requires the assembly of gene specific targeting constructs. Before the advent of recombineering^8^,^9^ this was often tedious and laborious. Recombineering greatly simplified the methods for rapid assembly of long homology arm (>4 kb each arm) targeting constructs^10^ and has been adapted for high-throughput production, resulting in more than 16,000 targeted mouse genes^11^.

Particularly since the advent of green fluorescent protein (GFP)^12^,^13^ protein tagging has empowered cell biology and functional genomics with fluent access to protein localization, live cell imaging and generic affinity purification for proteomics and chromatin immunoprecipitation amongst other applications. Although knock-in targeting into the endogenous gene is conceptually the best way to tag a chosen protein, to date the obstacles involved in achieving this optimum outcome have been circumvented by alternative approaches such as the use of fosmid and BAC transgenes^14^,^15^,^16^.

Because it is highly efficient, nuclease-assisted targeting not only enables gene targeting in many previously inaccessible genomes but could also expedite simultaneous homologous recombination on both alleles in diploid hosts^17^. Thereby, conditional loss-of-function strategies could be implemented in one step of simultaneous targeting.

Here our first aim was to develop simple and rapid methods for Cas9-assisted targeting for protein tagging. Our second aim was to evaluate whether Cas9 could be used to routinely achieve simultaneous biallelic targeting. Because biallelic targeting would open a variety of options, we explored the possibility that it could be achieved using CRISPR/Cas9 and applied to protein tagging and conditional mutagenesis.

## Results

### One-step gRNA expression plasmid construction

**Figure 1:**
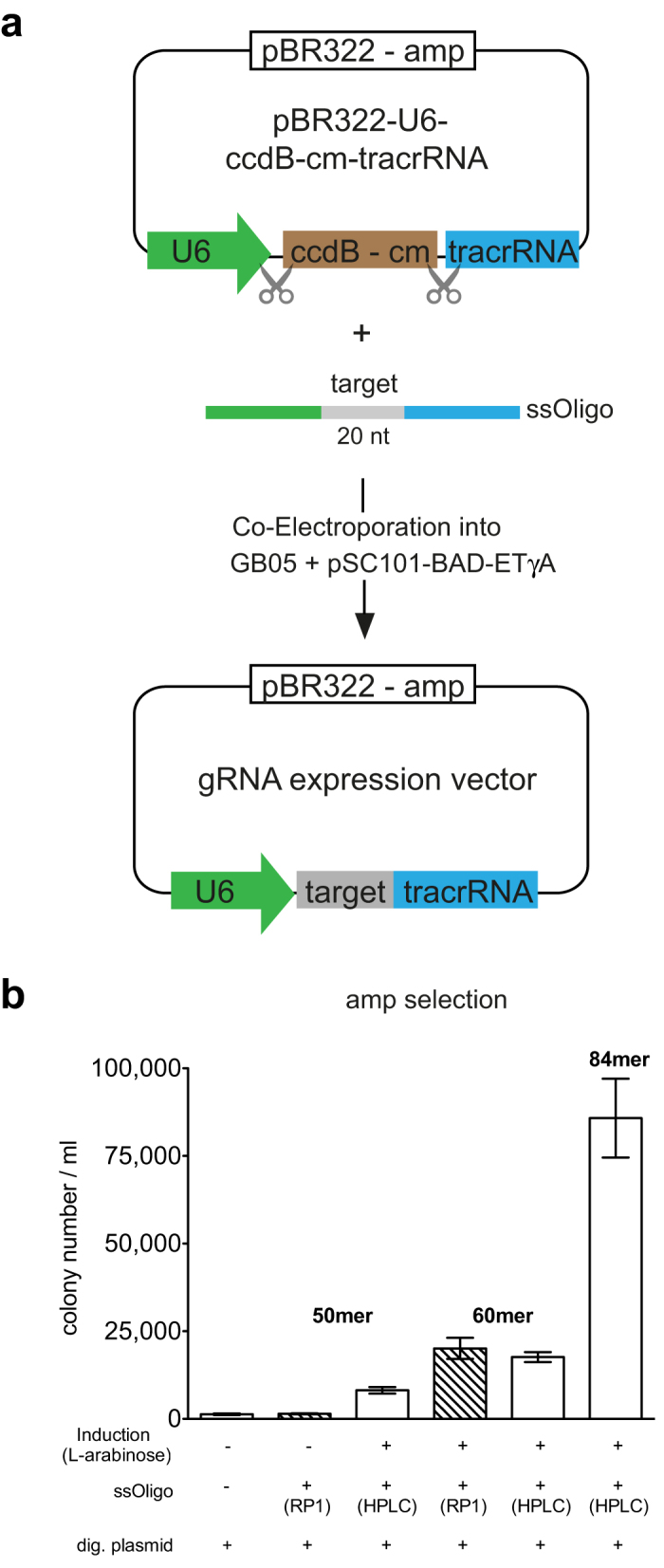
One-step generation of gRNA expression plasmids. (**a**) Scheme of the cloning strategy showing pBR322-U6-ccdB-cm-tracrRNA, which is digested with BstZ171 and NheI to release the ccdB-cm cassette and then co-electroporated with a single stranded oligonucleotide into GB05 after arabinose induction of pSC101-BAD-ETγA. The oligonucleotides contained the 20nt gRNA sequence flanked by variable lengths of sequence identity (i.e. homology boxes) to the U6 promoter and tracrRNA regions. (**b**) Ampicillin resistant colonies after co-electroporation of single stranded oligonucleotides each containing the same 20nt gRNA flanked by homology boxes of different lengths, either 14nt (50mer), 19nt (60mer) or 31nt (84mer). In addition the oligonucleotide included the GG dinucleotide required for the U6 promoter between the 5′ homology arm and the gRNA. Error bars, s.d. (n = 3). RP1 and HPLC-reverse phase or high performance liquid chromatography purifications of the oligonucleotides by the manufacturer. Six clones from each experiment were sequenced. From left to right, the correct cloned product was observed 0/6; 0/6; 3/6; 5/6; 6/6; 6/6 times respectively.

We applied a recent recombineering advance based on annealing recombination mediated by full-length RecE/RecT^18^ to establish a one-step method to generate guide RNA (gRNA) expression plasmids (Fig. 1a). An established U6 expression plasmid^6^ was modified by improving the tracrRNA sequence^19^ and incorporating the *ccdB* toxin gene^20^ at the gRNA cloning site. A batch of this plasmid was digested with BstZ171 and NheI, column purified and 200 ng aliquots mixed with 50 pmol oligonucleotides encoding 20 nucleotide gRNAs. The chosen 20nt gRNA sequence is flanked by homology arms (i.e. identical sequences) to sequences in the plasmid either side of the restriction cleavages (Supplementary Table 1). The gRNA sequences are incorporated into the pBR322-U6 expression plasmid upon co-electroporation into *E. coli* induced to express RecE/RecT. Due to its toxicity, the *ccdB* gene ensures no carryover of the parent plasmid so that virtually all ampicillin resistant colonies carry the gRNA insert. For economy, we tested different homology arm lengths to find that 19 bp each is sufficient, so that the single stranded oligonucleotides need only be 60mers. Also, they do not need to be HPLC purified (Fig. 1b). In a recent application, we generated 304 gRNA expression plasmids out of 312 attempts at the first go using 80 mer oligonucleotides and 96 well boxes in a simplified recombineering pipleine^21^. All 8 initial failures were obtained on the second go using newly synthesized oligonucleotides, indicating that oligonucleotide quality was the cause of the failure (M. Sarov, AFS; unpublished observations). Hence this one-step method to build gRNA expression plasmids is simple fast and cost-effective.

### One-step short-arm targeting construct

Potentially, an advantage of nuclease-assisted targeting lies with the employment of short-arm (~1 kb) targeting constructs because genotyping by junction PCR can be employed. This practical advantage is not available for conventional targeting in mouse ESCs because longer arms are required to achieve workable targeting efficiencies^1^. Because nuclease-assisted targeting is mechanistically different than conventional targeting, the advantages of short-arm targeting constructs are worth re-examination.

**Figure 2:**
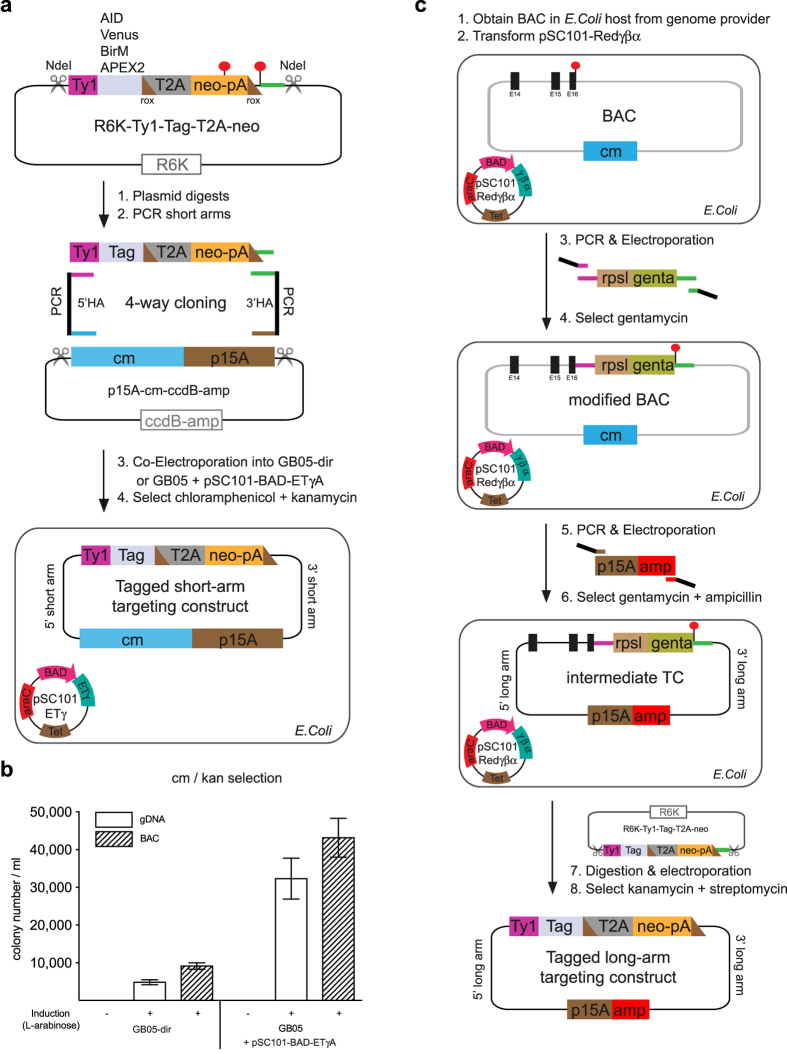
Two different recombineering methods to generate targeting constructs for protein tagging. (**a**) The 4-way recombination strategy mediated by full-length RecET to generate short arm targeting constructs is illustrated. The C-terminal tagging cassette consisting of the Ty1 linker, tag coding region (either AID, Venus, BirM or APEX2), T2A peptide and promoterless neomycin gene (neo) in R6K-Ty1-Tag-T2A-neo is released from its vector by NdeI digestion. The p15A origin and chloramphenicol (cm) cassette in p15A-cm-ccdB-amp is released by NdeI restriction digestion and mixed with 5′ and 3′ homology arms (HA) that were generated by PCR from genomic or BAC DNA. At their 5′ ends the PCR primers include 50nts of sequence homology to the respective ends of the cassettes indicated by the four colours (Supplementary Table 1). The mixture is electroporated into *E. coli* induced with arabinose to express RecE/RecT/Redγ from either the genome (strain GB05-dir) or an expression plasmid (pSC101-BAD-ETγA). Double selection for chloramphenicol and kanamycin resistance identifies the targeting vector. Neither of the parental plasmids will be selected because the R6K origin will not replicate in GB05 and ccdB counterselection blocks p15A-cm-ccdB-amp propagation. Brown triangles denote the 34 bp rox sites, red circles denote stop codons and pA is a polyadenylation signal. (**b**) Recombination efficiencies promoted by full-length RecE/RecT. The 4-way mixture illustrated in (**a**) based on Ash2l using the T2A version was electroporated into either arabinose induced GB05 containing pSC101-BAD-ETγA plasmid or GB05-dir, in which the PBAD-ETγA operon has been integrated into the genome. The homology arms were amplified from either genomic DNA (gDNA) or a BAC carrying the target gene (BAC). Correct recombinants were retrieved by selection for chloramphenicol (cm) and kanamycin (kan) resistance, followed by restriction mapping. In all four cases, at least 23/24 clones were correct. (**c**) Diagram of the Red recombineering method to generate long arm targeting constructs for C-terminal tagging. A BAC clone of the gene to be targeted is obtained from a genome provider and transformed with pSC101Redγβα. Oligonucleotides including 50nt homology either side of the chosen C-terminus in the BAC are attached to the rpsL-gentamycin cassette by PCR. After arabinose induction of Red operon expression, the PCR product is electroporated into the *E. coli* host. Selection for gentamycin resistance identifies the candidate recombinants. The rpsL-gentamycin cassette is flanked by the same standard homology arms illustrated in purple and green in (**a**). In the next step, PCR is used to attach 50nt homology boxes, which define the ~9 kb region to be copied from the BAC into the p15A-ampicillin subcloning vector. Selection for gentamycin and ampicillin resistance identifies the candidate subcloned intermediate targeting constructs. Protein tags from the R6K series can be readily introduced into these intermediate TCs using the standard purple and green homology boxes with selection for kanamycin and streptomycin resistance.

Furthermore using full-length RecE/RecT^18^, short arm targeting constructs can be built rapidly in one recombination step using a 4-way reaction (Fig. 2a). To complement this new application, we built a recombineering-ready plasmid series for C-terminal protein tagging. Coding regions for the auxin degron (AID^22^), the fluorescent protein Venus^23^ and the enzymes for biotin proximity labeling BirM^24^ and APEX2^25^,^26^ have been cloned into the same context in a plasmid series based on the R6K origin. The context includes on the 5′ side a flexible protein linker based on two copies of the Ty1 peptide, which serves two additional purposes. It is an epitope for the useful monoclonal antibody, BB2^27^ and its coding region serves as one of the standard 50 bp homology boxes for the recombineering reactions (coloured purple in Fig. 2; Supplementary Table 1). On the 3′ side the other standard 50 bp homology box is coloured green. In between the homology boxes reside the coding regions for the various protein tags and the neomycin (neo) antibiotic resistance gene for selection in one of two conformations. If the gene is expressed in the targeted cells, then the polycistronic strategy for selection using 2A peptides^28^ can be employed. If the gene is not expressed sufficiently for polycistronic selection, then the PGK promoter-driven strategy can be employed. In both cases, the neomycin gene includes an embedded prokaryotic promoter for expression of kanamycin resistance in *E. coli*^10^ and is flanked by rox sites for later removal by Dre recombinase^29^ if required. To utilize the tagging cassettes in recombineering, the plasmids are digested with NheI, which cuts at the outside ends of the homology arms. The plasmids are based on the R6K origin, which will not replicate in standard *E. coli* strains because they lack its cognate replication A protein, Pir^30^. Consequently the major source of unwanted recombineering background, which is carry-over of the template plasmid, is eliminated and the tagging cassette does not need to be gel purified after restriction digestion^31^,^21^.

To build the short-arm targeting constructs, the ~1 kb homology arms are amplified by PCR using either genomic DNA from the target cells, which is therefore isogenic, or BAC DNA carrying the target gene as the template. For PCR, the 60mer oligonucleotides include not only gene specific PCR primers at their 3′ ends but also the four standard 50 bp homology boxes to the tagging cassette (Fig. 2a; purple, green) and the subcloning vector (Fig. 2a; brown, blue). The subcloning vector is also prepared by restriction digest of p15A-cm-ccdB-amp. The presence of the *ccdB* toxin gene in the unwanted part of this plasmid ensures that only the p15A origin-chloramphenicol (cm) part will be propagated. The two restriction digests are together mixed with the two PCR products and co-electroporated into *E. coli* after arabinose induction of full-length RecE, RecT and Redγ expression from the BAD-ETγ operon either integrated into the *E. coli* chromosome (GB05-dir) or in pSC101-BAD-ETγA. Whether amplified from genomic or BAC DNA, or whether RecETγ was expressed from the *E. coli* chromosome or plasmid, virtually all colonies contained the intended 4-way recombination product upon double selection for chloramphenicol and kanamycin (Fig. 2b; Supplementary Figure 1a). Applying the same principles, a one-step, 5-way recombination reaction can be used to generate targeting constructs for loxP conditional alleles (Supplementary Figures 1b and 2). In our experience, failure to obtain the intended targeting construct is rare and almost always due to errors in oligonucleotide synthesis or PCR amplification.

### Recombineering for long-arm targeting constructs

Figure 2c illustrates the recombineering scheme for building long arm (~4 to 5 kb) C-terminal targeting constructs. In this application, the Red operon expression plasmid, pSC101Redγβα is introduced into an *E. coli* host carrying the selected BAC, which is then modified using a selectable/counterselectable cassette based on gentamycin and rpsL flanked by the same purple and green homology boxes employed in Fig. 2a. After recombineering into the chosen C-terminus, this region is then subcloned by recombineering^32^ to establish the intermediate targeting construct that can now be modified with any cassette from the R6K plasmid series.

### Implementing the auxin degron

Amongst the variety of possible examples, we present work with the auxin degron for two reasons. First, the auxin degron is a ligand-regulated conditional loss-of function strategy that requires the introduction of an effector protein, rice Tir1, which is an F-box protein that binds auxin and then conveys the auxin degron, AID (Auxin Inducible Degron; also known as Auxin/Indole 3-acetic acid or Aux/IAA repressor), to the SCF (Skp1, Cullin, F-box protein) E3 ubiquitin ligase complex for polyubiquitinylation and subsequent degradation by the proteasome^22^.

Second, for autosomal genes, both alleles need to be tagged (or tagged and mutagenized) to achieve inducible loss-of-function. Hence targeted tagging with the auxin degron requires more work to establish than tagging for fluorescent studies or proximity mapping. Our initial applications with rice Tir1 showed only partial depletion of nuclear proteins (data not shown). Hence we codon optimized Tir1 for mammalian expression, reduced the number of CpG dinucleotides, added a nuclear localization signal (nls; Supplementary Figure 3a) and targeted it to the Rosa26 locus with expression driven by the strong CAG promoter (Supplementary Figure 4). The AID coding region was similarly codon optimized (Supplementary Figure 3b).

### Comparing short- and long-arm Cas9 assisted targeting

**Figure 3:**
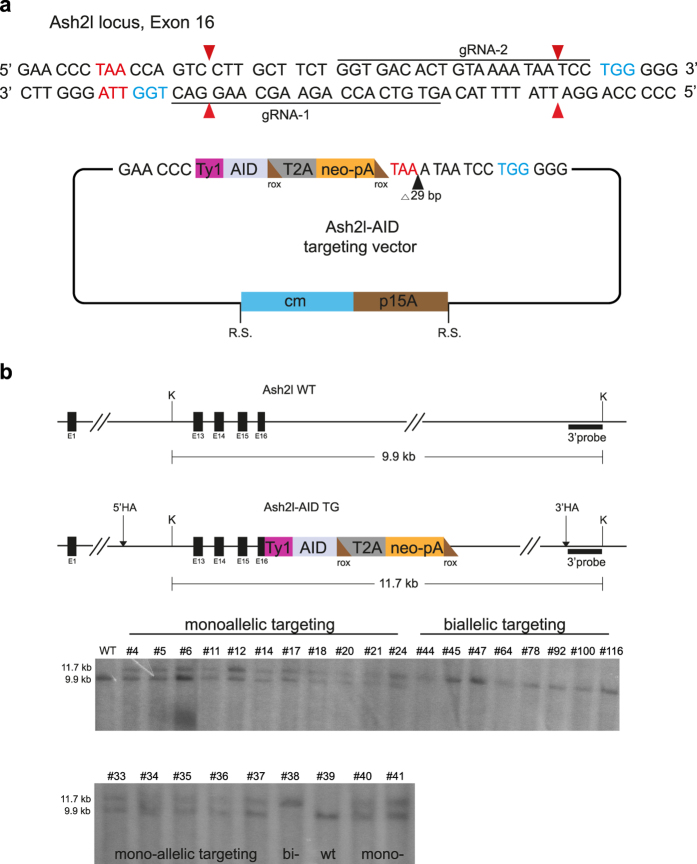
Cas9-assisted targeting at the C-terminus of Ash2l. (**a**) Ash2l sequence near its stop codon (highlighted in red) is shown with two chosen gRNAs and their corresponding Cas9 cleavage sites (red triangles, PAM sequences in blue). The Ash2l AID targeting constructs are also illustrated indicating the cassette fusion junctions and the 29 bp deletion to prevent Cas9 loaded with either gRNA 1 or 2 from cleaving the targeting construct. Rox sites (brown triangles), which do not interrupt the open reading frame, flank the neomycin selection cassette for optional later removal by Dre recombinase. In the subcloning step to establish the intermediate targeting construct, the p15A origin of replication and antibiotic resistance gene are flanked by unique restriction sites (R.S.) for digestion before targeting in ESCs. (**b**) Diagram of the Ash2l locus, Southern strategy and Southern analysis of DNA prepared from resistant Ash2l-AID-T2A-neo ESC colonies, digested with KpnI (K) and hybridized with the 3′ probe. Twenty eight different ESC candidates plus a wt control are shown in the two Southern panels.

Short-arm (~1 kb each arm) and long-arm (~4.5 kb each arm) targeting constructs were generated for targeting AID to the C-terminae of two genes, Ash2l and Wdr82, in the Rosa26-CAG-nlsTir1 ESCs. To ensure that Cas9 did not also cut the targeting construct, small gaps were introduced between the homology arms to omit parts of the gRNA target site. The strategy for Ash2l, which included the testing of two different gRNAs, is presented in Fig. 3. Targeting efficiency was scored for monoallelic and biallelic targeting (Table 1). High frequencies of monoallelic targeting were achieved with all four targeting constructs. As expected, most monoallelically targeted ESCs showed Cas9-induced damage on the other allele (10/14; Supplementary Figure 5). Consequently the status of the second allele must be evaluated. With this precaution, the advantages of short-arm targeting constructs can be readily applied to monoallelic applications such as tagging with a fluorescent protein, targeting the sex chromosomes in male cells or heterozygous targeting to generate a mouse. In addition to Ash2l and Wdr82, to date we have successfully monoalleically tagged 11 (of 11) genes in ESCs (AG, MO, Davi Coe Torres, KA, AFS; unpublished observations).

For simultaneous biallelic targeting, a different outcome was found. Whereas both Ash2l and Wdr82 were biallelically targeted using long-arm targeting constructs, thereby demonstrating that C-terminal tagging of both proteins with AID was not lethal in ESCs, neither gene was biallelically targeted with the short-arm constructs (Table 1). Notably, the frequencies of monoallelic targeting with short-arm constructs, although high, were also lower than the very high frequencies achieved with long-arm constructs. Although the convenience of short-arm targeting constructs, both from their ease of construction and ease of PCR genotyping, can be productively applied for monoallelic targeting, we conclude that longer homology arms deliver an important increase in efficiency for applications that require biallelic targeting. To explore this issue further, we generated a targeting construct with intermediate length homology arms (1419 and 1509 bps) by digestion of the Ash2l long-arm targeting construct with AseI and BglI. Again monoallelic targeting frequencies were high (Table 1; 69%) and notably a few biallelic events were found (3/70; 4%). These intermediate length homology arms therefore delivered an outcome intermediate between the short- and long-arm targeting constructs.

### Functional evaluation of auxin degron

**Figure 4:**
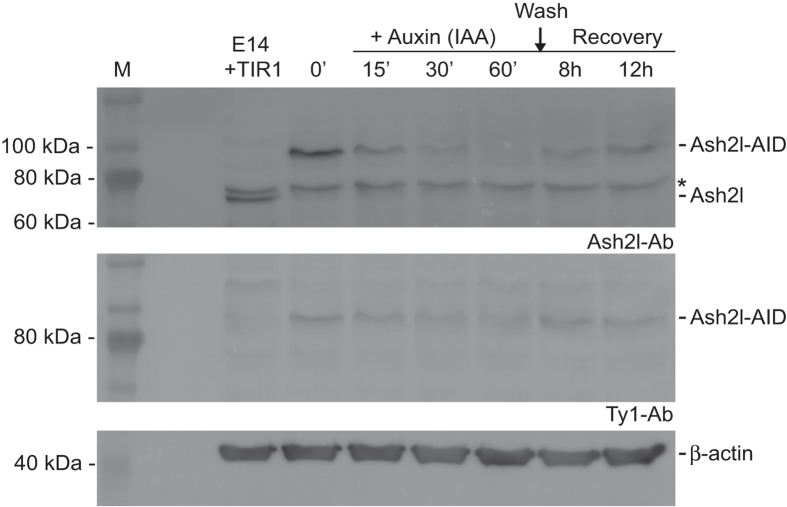
Protein degradation promoted by auxin. Western blot of Ash2l-AID protein (68 kDa + 33 kDa = 101 kDa) using an Ash2l antibody (top panel) and the BB2 monoclonal antibody against Ty1 (middle panel). Cells expressing nlsTir1 and biallelically targeted Ash2l-AID were treated with 500 μM indole-3-acetic acid (IAA) for the indicated times. For protein recovery, cells were washed after 60 minutes of IAA treatment and cultured in ES medium without auxin for 8 and 12 hours. Untargeted E14Tg2a cells expressing Tir1 was the negative control (left hand column) and β-actin as loading control (lower panel). The Ash2l antibody detects a non-specific band marked with an asterisk.

As evaluated using both an Ash2l antibody and the Ty1 monoclonal antibody, administration of auxin (indole-3-acetic acid) in the *rosa26 tir1/*^+^, *ash2l-AID/ash2l-AID* biallelically targeted ESCs resulted in rapid loss of the Ash2l-AID fusion protein, which slowly recovered after auxin removal (Fig. 4) thereby functionally validating the targeting and the auxin degron strategy in ESCs.

## Discussion

We developed RAC-tagging to apply the potential of CRISPR/Cas9 to protein tagging. For monoallelic tagging, RAC-tagging couples the genotyping advantage of junction PCR for short-arm targeting with a very rapid method to build the targeting constructs. For biallelic targeting, our results indicate that long-arm targeting constructs promote the successful outcome. Whether using short- or long-arm constructs, multiple reuse of a gene-specific reagent to introduce a different tag from the R6K series is straightforward because the RAC-tagging system is modular by design. To this end, we are expanding the tagging repertoire by introducing more protein tags into the R6K series.

Recently a generic, homology-independent strategy for monoallelic Cas9-assisted gene tagging was reported, which needs only the gene-specific gRNA and circumvents the need to build gene-specific targeting constructs^33^. This method is based on ligation of the tagging cassette into the Cas9 genomic cleavage and consequently the intended event is one possibility amongst a variety of different events promoted by the double strand break. The correct clones therefore have to be identified amongst a majority of unwanted events, which include imperfectly ligated junctions. We show here that the gene-specific targeting constructs can be rapidly built and bring the advantage that the majority of clones are correctly targeted.

Amongst the various tags available, we present our experience with the auxin degron because it is relatively unexploited and has great potential for functional studies. As illustrated before^22^,^34^ and in Fig. 4, target protein removal can be remarkably rapid. In vertebrate systems, this speed opens new opportunities for real-time functional studies that have been previously inaccessible using currently available loss-of-function strategies such as RNAi knock-down or Cre-mediated site-specific recombination.

Like the widely used gene switch based on ligand-regulated site-specific recombination^35^, the auxin degron benefits from the advantages of ligand administration to trigger ablation of the target gene product. Both these ligand-inducible strategies require the expression of an effector protein, either rice Tir1 or an SSR-LBD (site specific recombinase/ligand binding domain fusion protein), usually CreERT2^36^. However the auxin degron offers two advantages. First, as opposed to the genetic deletion effected by site-specific recombination, protein ablation by the auxin degron is reversible. Second, protein ablation by auxin is significantly faster than other loss-of-function methods. These advantages suggest that the auxin degron can bring greater accuracy and flexibility to functional studies. However the AID protein tag used here and before^22^,^34^ is the full-length Aux/IAA repressor, which can homodimerize^37^. Reduction of AID to the core 46 amino acids needed for binding to auxin-bound Tir1 without the dimer interface has been reported to work as well as full-length AID in yeast^38^. However this is not the case in ESCs where the core 46 amino acids does convey auxin-dependent degradation but not as efficiently as full-length AID (Supplementary Figure 6). This indicates the need for sequences N-terminal to the core 46 amino acids, which contribute to the auxin response in plants and include the N-terminal part of the bipartite AID nuclear localization sequence^39^. Although successful application to cytoplasmic proteins has been described^34^, some consideration of the potential complications arising from unwanted dimerization and/or nuclear localization appears advisable at this stage.

## Methods

### DNA constructs

For the 4-way and 5-way recombinations, R6K-Ty1-Tag-neo and p15A-cm-ccdB-amp plasmids were restricted with NdeI and purified using Invisorb Fragment CleanUp Kit (Stratec). The R6K plasmids were grown in EC100D-pir116 (Epicenter). The 5′ and 3′ homology arms were amplified from either genomic or BAC DNA using High-Fidelity polymerase and primer pairs that contained 50 bases of sequence homology to the respective ends of the cassettes (Supplementary Table 1). All four or five DNA fragments (each 500 ng) were co-electroporated into cells induced with L-arabinose to express RecE/RecT/Redγ^18^ and then plated on LB plates containing chloramphenicol (15 μg/ml) and kanamycin (10 μg/ml). E14Tg2a genomic DNA or bacterial artificial chromosomes (BACs) containing Ash2l (bMQ-333I23) or Wdr82 (CH29-51445) were used to provide the homology arms.

The p15A-cm-ccdB-amp and pBR322-U6-ccdB-cm-tracrRNA plasmids were grown in a ccdB-resistant *E. coli* strain^40^. After restriction digestion with BstZ171 and NheI to release the *ccdB-cm* cassette, pBR322-U6-ccdB-cm-tracrRNA was purified. Linearized plasmid (200 ng) and 50 pmol of single stranded oligonucleotide were co-electroporated into RecET bacteria as described above and plated on LB plates containing ampicillin (100 μg/ml). Only cells that carry the plasmid with oligonucleotide without ccdB expression survive. For the mouse Ash2l and Wdr82 genes, target sites close to endogenous stop codon with the sequence 5′-N_20_NGG were selected.

For recombineering methods to generate long-arm targeting constructs, please see^15^,^41^.

*Arabidopsis* IAA17 degron (AID) and rice Tir1 were codon-optimized for mammalian expression by Genescript GmbH after manual elimination of CpG dinucleotides. An N-terminal nuclear localization signal (nls) was added to Tir1. Nls-TIR1 and AID were recombineered into a Rosa26-CAG-IRES-puro targeting vector and pR6K-Ty1-T2A-neo respectively. Nls-FLAG-linker-Cas9, optimized for nuclear import of Cas9^42^,^43^, was obtained from pST1374 plasmid (Addgene; #13426) and cloned into pCAG-IRES-puro. gRNA-AAV1-T2 (Addgene; #41818) was modified to create pBR322-U6-ccdB-cm-tracrRNA by recombineering to insert a ccdB-cm cassette flanked by BstZ171 and NheI restriction sites in place of the 20 bp target sequence. Plasmid sequence files can be obtained from our website http://www.biotec.tu-dresden.de/research/stewart/group-page/plasmids.html.

### Cell culture

E14Tg2a embryonic stem cells were maintained in Dulbecco’s modified Eagle’s Medium (DMEM) supplemented with 15% fetal calf serum (FCS), 2 mM L-glutamine, 1 mM sodium pyruvate, 1% penicillin/streptomycin, 100 μM non-essential amino acids (all from Invitrogen), 100 μM β-mercaptoethanol (Sigma) containing leukemia inhibitory factor (LIF) in the presence of 1 μM PD-0325901 and 3 μM CT-99021 (“2i”) at 37 °C with 5% CO_2_. ESCs (5 × 10^6^) were electroporated with 40 μg linearized targeting vector (Rosa26-CAG-NLS-TIR1-IRES-puro) or co-electroporated with 20 μg linearized targeting vector (Ash2l-, Wdr82-AID), 15 μg gRNA plasmid and 5 μg NLS-FLAG-linker-Cas9 expression vector using a Bio-Rad electroporator (250 V, 500 μF) and selected for 7 days with 1 μg/ml puromycin (Sigma) or with 200 μg/ml G418 (Invitrogen), respectively. Double targeted ES cells were expanded under double selection (puromycin + G418) and induced with 500 μM indole-3-acetic acid (IAA, Sigma).

### Western blot

ES cells were sonicated in 20 mM HEPES pH 8.0; 150 mM NaCl; 1.5 mM MgCl_2_; 10% glycerol; 2 mM EDTA pH 8.0; 0.05% Tween-20) with 1 mM DTT and 1xprotease inhibitor cocktail (Roche) using a BioRaptor waterbath (Diagenode). Whole cell extracts were separated by SDS-PAGE (10% Tris-glycine) transferred to PVDF membranes and probed with primary antibodies: rabbit anti-Ash2l IgG (1:2000; A300-112A, Bethyl), mouse anti-Ty1 IgG (1:5000; home made), mouse anti-β-actin IgG (1:5000; Sigma) overnight at 4 °C, then incubated with goat anti-rabbit (1:30,000)/mouse (1:5000; Sigma) horseradish-peroxidase conjugated secondary antibody for 1 h at RT and visualized with Supersignal West Pico substrate kit by Luminescent Image Analyzer LAS-4000 (Fujifilm Life Science).

### Genotyping

The frequencies of long-arm targeting reported in Table 1 were obtained from Southern blots as illustrated in Fig. 3 using standard procedures. The frequencies of the other targetings reported in Table 1 were achieved by PCR using standard procedures. The primers used for PCR genotyping and to generate the Southern probes are listed in Supplementary Table 1.

## Additional Information

**How to cite this article**: Baker, O. *et al*. RAC-tagging: Recombineering And Cas9-assisted targeting for protein tagging and conditional analyses. *Sci. Rep*. **6**, 25529; doi: 10.1038/srep25529 (2016).

## Supplementary Material

Supplementary Information

## Figures and Tables

**Table 1:** Comparison of targeting efficiencies using short- and long-arm targeting constructs.

	Ash2l (gRNA-1)	Ash2l (gRNA-2)	Wdr82
Monoallelic short-arm	71% 53/74	–	84% 141/167
Biallelic short-arm	0% 0/74	–	0% 0/167
Monoallelic long-arm	90% 97/108	90% 62/69	96% 83/86
Biallelic long-arm	7% 8/108	4% 3/69	1% 1/86
Monoallelic 1419/1509	69% 48/70	–	–
Biallelic 1419/1509	4% 3/70	–	–

Targeting efficiencies from Cas9 assisted targeting using either Ash2l gRNA 1 or 2 (Fig. 3) or Wdr82 gRNA employing targeting constructs with different homology arm lengths (short; ~1 kb; long; ~4.5 kb; or intermediate 1419 bp and 1509 bp) are presented as percentage success followed by the number of positive colonies divided by the total number of colonies successfully genotyped.
