# Supplementary material for: RAC-tagging: Recombineering And Cas9-assisted targeting for protein tagging and conditional analyses
Source: Sci Rep. 2016 May 24;6:25529. doi: 10.1038/srep25529 (PMC4877586; doi:10.1038/srep25529)
Supplement: Supplementary Information [file srep25529-s1.pdf]

Supplementary material for –

RAC-tagging: Recombineering And Cas9-assisted targeting for protein tagging and conditional analyses

Oliver Baker, Ashish Gupta, Mandy Obst, Youming Zhang, Konstantinos Anastassiadis, Jun Fu & A. Francis Stewart

Supplementary Figure 1. Mini-prep restriction analysis of short-arm targeting constructs.

a) A typical four-way recombination example (to target Ash2l with Venus) using the method depicted in Figure 2a. The gel shows 21/24 were correct.

b) A typical five-way recombination example (to generate a multipurpose loxP conditional allele with lacZ reporter for *Mll4*). The gel shows 18/24 correct with two partial digests (asterisks).

Supplementary Figure 2. One-step generation of conditional targeting constructs

a) 5-way cloning strategy. A standard knock-out first cassette [43] consisting of a β-galactosidase (LacZ) neomycin fusion gene (neo), polyA signal (pA), flanking FRT and prospective 5' loxP sites was released by restriction digestion from an R6K plasmid. Similarly p15A-cm-ccdB-amp was digested as in Figure 2a. Three ~1kb PCR products were generated from either genomic (gDNA) or BAC DNA. The 5' homology arm (HA) was generated in the same way as 4-way cloning (Figure 2). The 3' HA was generated in two pieces so that the 3' loxP site could be included in one of the PCR primer oligonucleotides. This oligonucleotide also included 40 nucleotides of sequence identity to the other half of the 3'HA. As for the knock-out first strategy, the two loxP sites are positioned either side of a critical exon (c.e.), which is in the PCR amplified region.

b) Recombination efficiencies promoted by full-length RecE/RecT. The methods are the same as 4-way cloning except that the five linear substrates were co-electroporated into GB05 containing pSC101-BAD-ETγA plasmid. Correct recombinants were retrieved by selection for chloramphenicol (cm)

and kanamycin (neo) resistance. Error bars, s.d. (n=3).

Supplementary Figure 3. Codon optimized AID and TIR1.

Sequence alignment of wild type (wt) and codon-optimized, CpG dinucleotide reduced,

a) rice Tir1 with N-terminally added nuclear localization signal;

b) auxin inducible degron (AID). Nucleotide changes are highlighted.

Supplementary Figure 4. Targeting nlsTIR1 to the Rosa26 locus

E14TG2a cells were electroporated with Rosa26-CAG-nls-Tir1-IRES-puro for targeting at the ubiquitously expressed Rosa26 locus.

a) The targeting strategy and Southern analysis of DNA prepared from resistant ES colonies, digested with *BamHI* (B) and hybridized with the 5′ probe. **b)** Northern blot of two independent targeted clones to validate nlsTir1 expression. E14TG2a wt cells served as a negative control. EtBr-stained 18S/28S rRNAs serve as loading control. **c)** Proliferation curves. Cells ($1x10^5$) were seeded on gelatine-coated plates and cultured in the presence or absence of 500µM IAA for 4 days. Cell number was determined every 24 hours. Error bars, s.d. (n=3).

Supplementary Figure 5. Cas9 cleavage at both Ash2l alleles

The 3' end of the Ash2l coding region was PCR amplified and sequenced from fourteen monoallelically targeted Ash2l-AID ES cell clones. Four clones showed no mutagenesis whereas the other ten showed the events depicted.

Supplementary Figure 6. Impaired function of shortened compared to full length AID when targeted onto the C-terminus of Ash2l in ESCs

The minimal 46 amino acid version of *Arabidopsis* Aux/IAA17 described by [35] was targeted to the C-terminus of Ash2l in R26-CAG-Tir1 ESCs reusing the Ash2l long arm targeting construct. Biallelicly full length and 46 amino acid targeted clones were compared by parallel culture and auxin inductions for the indicated times. The Western was incubated with the Ty1 monoclonal antibody. M – molecular weight marker; C – untargeted R26-CAG-Tir1 ESCs. β-actin served as the loading control.

Supplementary Figure 1

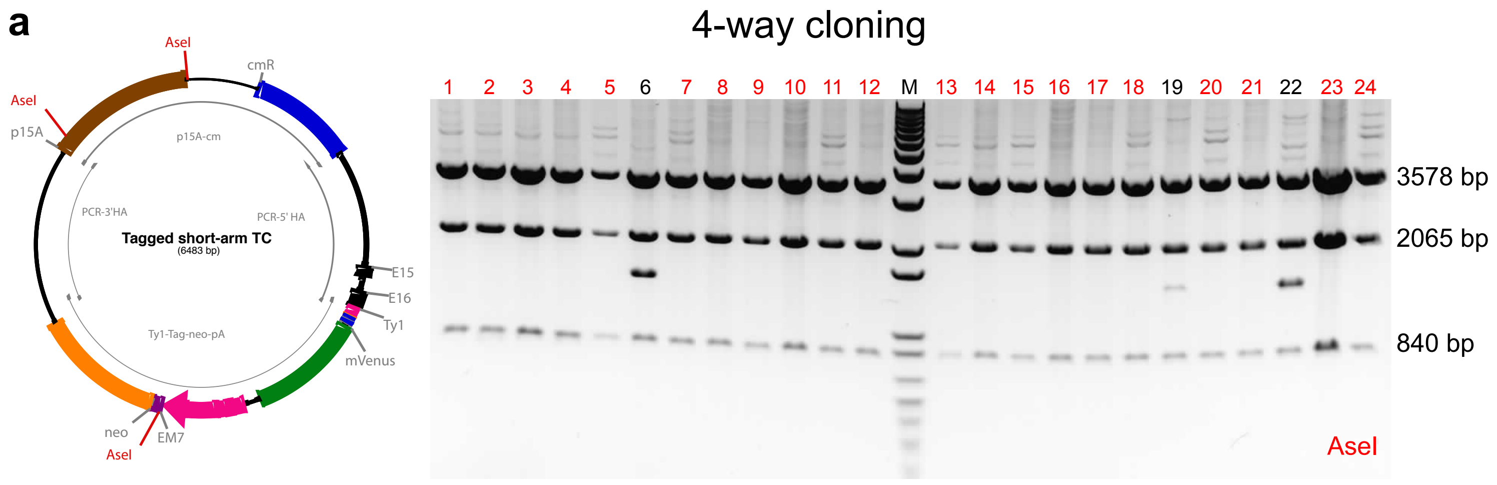

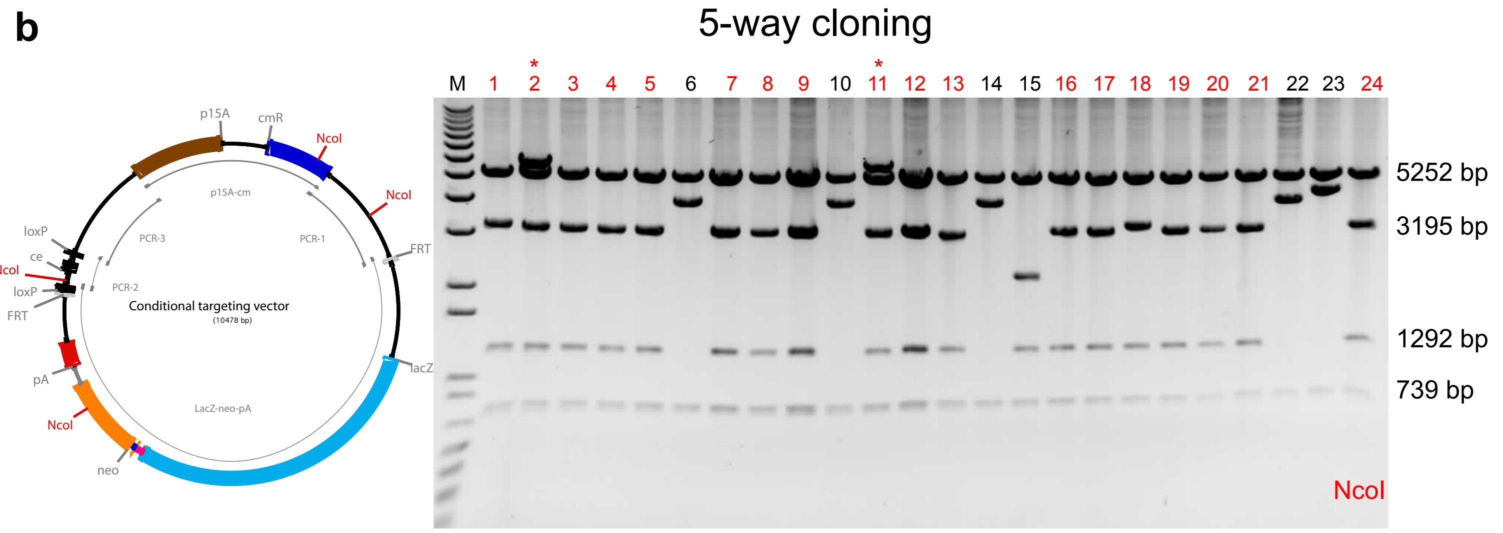

Supplementary Figure 2

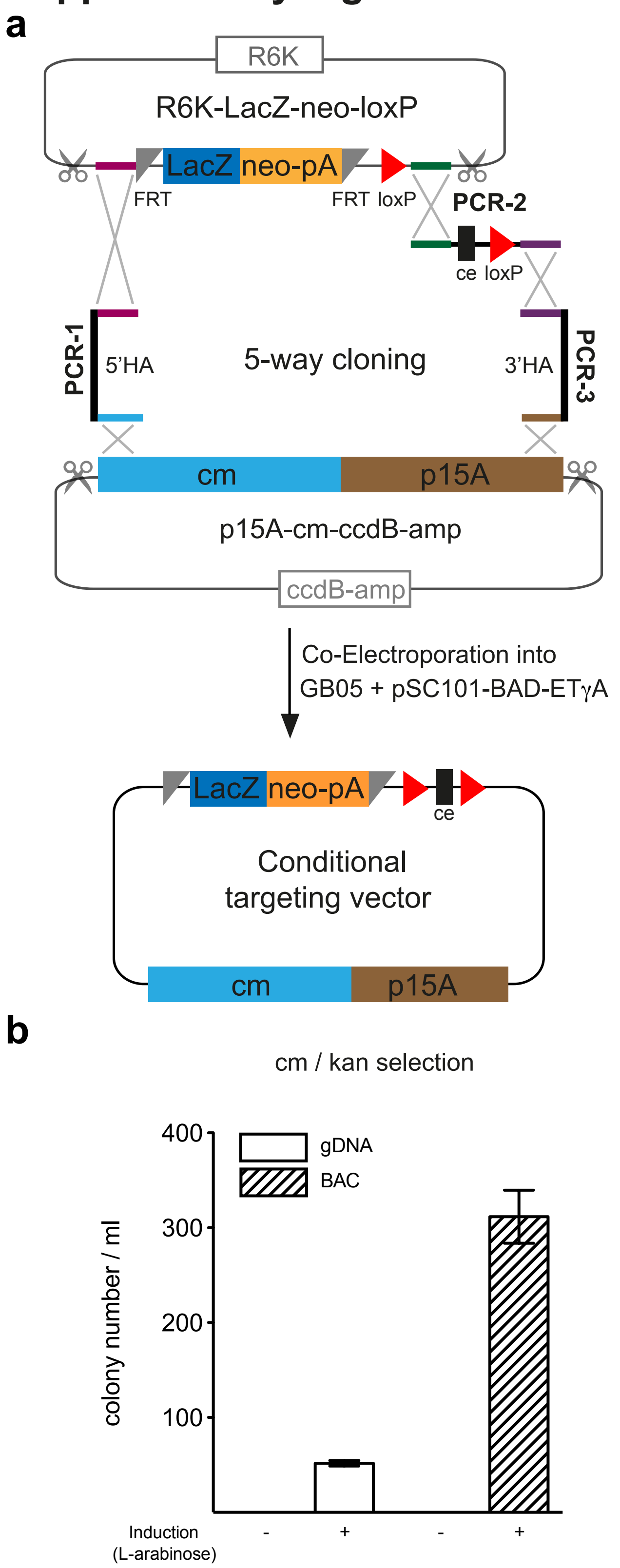

Supplementary Figure 3

nlsTIR1

```
              wt ATG --- --- --- --- --- --- --- --- --- --- --- --- --- ACG TAC TTC CCG 15
codon-optimized ATG GGC CCC GCC GCC CCC AAG AAG AAG AGA AAG GTG CTG GAC ACG TAC TTC CCT 54
              wt GAG GAG GTG GTG GAG CAC ATC TTC AGC TTC CTG CCG GCG CAG CGC GAC CGC AAC 69
codon-optimized GAA GAA GTT GTG GAA CAT ATC TTT TCT TTT CTT CCT GCC CAA AGA GAC CGG AAT 108
              wt ACG GTC TCG CTC GTC TGC AAG GTG TGG TAC GAG ATC GAG AGG CTG AGC CGC CGC 123
codon-optimized ACA GTC TCT TTG GTA TGT AAG GTT TGG TAT GAG ATT GAA AGG TTG TCA AGG AGA 162
              wt GGC GTC TTC GTG GGC AAC TGC TAC GCC GTG CGC GCC GGC CGC GTC GCC GCG CGG 177
codon-optimized GGA GTG TTT GTG GGA AAC TGC TAT GCG GTA AGG GCT GGA AGA GTC GCT GCC AGA 216
              wt TTC CCC AAC GTG CGG GCG CTC ACG GTG AAG GGG AAG CCC CAC TTC GCC GAC TTC 231
codon-optimized TTC CCC AAT GTG AGG GCA CTT ACA GTT AAG GGT AAA CCA CAT TTT GCT GAT TTC 270
              wt AAC CTC GTG CCC CCC GAC TGG GGC GGC TAC GCG GGG CCG TGG ATC GAG GCG GCC 285
codon-optimized AAT TTG GTT CCT CCG GAC TGG GGA GGT TAT GCT GGA CCA TGG ATT GAG GCT GCT 324
              wt GCG AGG GGA TGC CAC GGC CTG GAG GAG CTC AGG ATG AAG CGG ATG GTG GTG TCC 339
codon-optimized GCC AGA GGT TGT CAT GGG TTG GAG GAG TTG AGG ATG AAG AGG ATG GTT GTG TCT 378
              wt GAC GAG AGC CTC GAG CTG CTG GCT CGC TCG TTC CCG CGG TTC AGG GCT CTT GTT 393
codon-optimized GAT GAG TCC CTT GAA CTT CTG GCC CGG TCC TTC CCC AGA TTC AGA GCT TTG GTC 432
              wt CTT ATC AGC TGC GAG GGG TTC AGC ACT GAC GGG CTA GCC GCC GTC GCG AGC CAT 447
codon-optimized TTG ATT TCT TGT GAG GGT TTC TCT ACT GAC GGA CTC GCT GCA GTC GCT TCC CAC 486
              wt TGC AAG CTT CTG AGG GAG TTG GAT TTG CAG GAA AAT GAA GTG GAG GAT CGA GGG 501
codon-optimized TGT AAG TTG TTG AGG GAG TTG GAC CTC CAA GAA AAT GAG GTT GAG GAC AGA GGA 540
              wt CCT AGG TGG CTT TCC TGC TTC CCT GAT TCC TGC ACA TCA CTT GTC TCA TTG AAT 555
codon-optimized CCA AGA TGG TTG TCA TGT TTC CCT GAC TCA TGT ACT TCC CTT GTT TCC TTG AAT 594
              wt TTT GCC TGC ATC AAA GGG GAG GTT AAT GCT GGT TCA CTG GAG AGA CTT GTT AGC 609
codon-optimized TTC GCG TGT ATC AAA GGA GAG GTC AAT GCC GGG AGT CTT GAG AGA TTG GTC TCA 648
              wt AGG TCC CCA AAC CTG CGG AGT TTG AGG CTG AAT CGA TCT GTA TCG GTA GAT ACA 663
codon-optimized AGA AGT CCC AAC TTG CGA TCA CTG AGG CTC AAC AGA TCT GTG AGT GTG GAT ACC 702
              wt CTT GCA AAG ATA CTA CTG CGT ACC CCT AAC TTG GAG GAT TTG GGG ACA GGG AAT 717
codon-optimized TTG GCA AAG ATT CTC TTG AGA ACC CCC AAT TTG GAG GAC CTT GGA ACT GGG AAT 756
              wt TTG ACA GAT GAC TTC CAA ACT GAG TCC TAC TTT AAG CTT ACC AGT GCT CTG GAG 771
codon-optimized CTC ACG GAT GAC TTC CAA ACT GAG TCT TAC TTT AAG TTG ACT AGT GCT TTG GAA 810
              wt AAA TGC AAG ATG TTG AGG AGT TTG TCT GGA TTC TGG GAT GCT TCT CCT GTT TGC 825
codon-optimized AAG TGT AAG ATG TTG AGA TCA CTG TCC GGA TTC TGG GAT GCT TCA CCT GTT TGT 864
              wt CTG TCA TTT ATC TAC CCC CTG TGT GCT CAA CTG ACA GGA TTG AAC TTG AGC TAT 879
codon-optimized CTT TCC TTC ATC TAC CCT CTT TGT GCC CAG CTG ACT GGA CTC AAC TTG TCC TAT 918
              wt GCC CCC ACA CTT GAT GCT TCT GAC CTT ACA AAA ATG ATT AGC CGC TGT GTG AAG 933
codon-optimized GCT CCA ACA CTG GAT GCG AGT GAC TTG ACC AAG ATG ATT TCA AGA TGC GTG AAG 972
              wt CTC CAA CGC CTT TGG GTA CTG GAT TGT ATC TCG GAC AAA GGC TTG CAA GTG GTG 987
codon-optimized CTT CAA AGA CTT TGG GTC CTC GAT TGT ATC TCC GAC AAA GGA CTT CAA GTG GTG 1026
              wt GCC TCC AGT TGC AAA GAC TTG CAA GAA CTC AGG GTA TTT CCA TCA GAT TTC TAC 1041
codon-optimized GCA TCA TCT TGT AAG GAT CTC CAA GAG TTG AGA GTG TTT CCA AGT GAC TTC TAT 1080
              wt GTA GCT GGT TAT TCT GCA GTG ACA GAG GAG GGA CTT GTT GCA GTA TCC TTG GGC 1095
codon-optimized GTG GCA GGT TAC AGT GCT GTA ACT GAG GAA GGG TTG GTT GCT GTC TCC CTT GGA 1134
              wt TGT CCA AAA CTG AAC TCA CTA CTG TAC TTC TGT CAC CAA ATG ACT AAT GCT GCA 1149
codon-optimized TGT CCC AAG TTG AAT TCA CTC TTG TAC TTT TGT CAC CAA ATG ACT AAC GCT GCC 1188
              wt CTA GTT ACT GTC GCC AAG AAC TGT CCA AAT TTC ACA CGA TTC AGA CTT TGT ATT 1203
codon-optimized CTT GTG ACA GTG GCC AAA AAT TGC CCT AAC TTC ACA CGC TTC AGA CTT TGT ATC 1242
              wt CTT GAG CCA GGG AAG CCT GAT GTT GTG ACA AGC CAA CCA TTA GAT GAA GGC TTT 1257
codon-optimized CTT GAA CCT GGG AAA CCT GAC GTG GTC ACC AGT CAG CCC TTG GAC GAA GGA TTT 1296
              wt GGA GCT ATT GTT CGT GAG TGC AAG GGA TTA CAA CGT TTG TCA ATA TCT GGT CTT 1311
codon-optimized GGG GCG ATT GTG AGA GAA TGT AAG GGA CTC CAA AGA CTC TCT ATC AGT GGA CTC 1350
              wt CTC ACA GAC AAA GTT TTC ATG TAT ATT GGG AAA TAT GCA AAA CAA CTT GAG ATG 1365
codon-optimized CTG ACT GAC AAG GTG TTT ATG TAC ATT GGT AAG TAC GCT AAG CAG CTG GAA ATG 1404
              wt CTT TCT ATA GCA TTT GCT GGT GAC AGT GAT AAG GGT ATG ATG CAT GTT ATG AAT 1419
codon-optimized CTG TCT ATT GCC TTT GCC GGT GAT TCT GAC AAG GGG ATG ATG CAT GTT ATG AAT 1458
              wt GGA TGC AAG AAT TTA AGG AAA CTG GAG ATA AGA GAT AGC CCG TTT GGT GAT GCT 1473
codon-optimized GGA TGT AAG AAC TTG AGA AAG CTT GAG ATA AGA GAC AGT CCA TTC GGA GAT GCA 1512
              wt GCA CTC TTG GGG AAT TTT GCT AGG TAC GAG ACA ATG CGA TCC CTT TGG ATG TCA 1527
codon-optimized GCC CTC CTT GGT AAC TTC GCA AGA TAC GAA ACT ATG AGG TCT CTT TGG ATG TCC 1566
              wt TCT TGC AAT GTC ACG TTA AAG GGG TGC CAA GTC CTT GCG TCA AAG ATG CCG ATG 1581
codon-optimized AGC TGT AAT GTT ACA CTC AAG GGA TGT CAA GTG CTG GCC TCA AAA ATG CCA ATG 1620
              wt CTC AAT GTT GAG GTC ATA AAT GAG CGG GAT GGT AGC AAT GAA ATG GAG GAA AAC 1635
codon-optimized CTG AAT GTG GAG GTG ATC AAC GAG AGA GAT GGA TCC AAT GAA ATG GAA GAG AAC 1674
              wt CAT GGA GAT CTG CCT AAA GTG GAG AAA TTA TAT GTG TAC CGC ACA ACT GCT GGG 1689
codon-optimized CAC GGA GAT CTT CCC AAG GTG GAA AAG TTG TAT GTC TAC AGA ACC ACT GCT GGG 1728
              wt GCG AGG GAT GAT GCA CCA AAT TTT GTT AAA ATC CTA 1725
codon-optimized GCA AGG GAT GAC GCT CCA AAC TTC GTG AAA ATA CTG 1764
```

AID

```
              wt ATG ATG GGC AGT GTC GAG CTG AAT CTG AGG GAG ACT GAG CTG TGT CTT GGT CTT CCC 57
codon-optimized ATG ATG GGA TCA GTG GAA CTC AAC TTG AGG GAA ACG GAA TTG TGT CTG GGA CTT CCT 57
              wt GGT GGA GAT ACA GTG GCT CCG GTA ACC GGA AAC AAG AGA GGG TTC TCA GAG ACG GTT 114
codon-optimized GGG GGA GAT ACA GTT GCA CCA GTG ACT GGA AAC AAA AGA GGT TTC TCT GAA ACA GTG 114
              wt GAT CTG AAG CTA AAT CTG AAT AAT GAG CCT GCA AAC AAG GAA GGA TCT ACG ACT CAT 171
codon-optimized GAT CTG AAG CTG AAC CTT AAC AAT GAA CCT GCT AAC AAG GAG GGA TCC ACC ACC CAT 171
              wt GAC GTC GTG ACT TTT GAT TCC AAG GAG AAG AGT GCT TGT CCT AAA GAT CCA GCC AAA 228
codon-optimized GAT GTA GTA ACA TTC GAT TCT AAA GAA AAA TCT GCC TGT CCC AAA GAT CCA GCC AAG 228
              wt CCT CCG GCC AAG GCA CAA GTT GTG GGA TGG CCA CCG GTG AGA TCA TAC CGG AAG AAC 285
codon-optimized CCC CCA GCC AAG GCC CAA GTG GTG GGG TGG CCC CCA GTA AGG TCC TAC CGC AAG AAT 285
              wt GTG ATG GTT TCC TGC CAA AAA TCA AGC GGT GGC CCG GAG GCG GCG GCG TTC GTG AAG 342
codon-optimized GTC ATG GTC AGT TGT CAA AAG TCC TCT GGT GGA CCT GAA GCA GCC GCT TTC GTG AAA 342
              wt GTA TCA ATG GAC GGA GCA CCG TAC TTG AGG AAA ATC GAT TTG AGG ATG TAT AAA AGC 399
codon-optimized GTG TCA ATG GAT GGA GCT CCA TAC CTG AGA AAG ATT GAC CTG AGA ATG TAC AAG TCT 399
              wt TAC GAT GAG CTT TCT AAT GCT TTG TCC AAC ATG TTC AGC TCT TTT ACC ATG GGC AAA 456
codon-optimized TAT GAC GAG CTG AGT AAT GCT CTC AGT AAT ATG TTC TCC TCT TTC ACA ATG GGA AAA 456
              wt CAT GGA GGA GAA GAA GGA ATG ATA GAC TTC ATG AAT GAG AGG AAA TTG ATG GAT TTG 513
codon-optimized CAT GGG GGT GAA GAA GGT ATG ATT GAT TTT ATG AAT GAG AGA AAG CTT ATG GAT CTG 513
              wt GTG AAT AGC TGG GAC TAT GTT CCC TCT TAT GAA GAC AAA GAC GGT GAT TGG ATG CTC 570
codon-optimized GTT AAC TCC TGG GAT TAT GTT CCC TCC TAC GAG GAC AAA GAC GGA GAC TGG ATG TTG 570
              wt GTC GGC GAC GTT CCT TGG CCA ATG TTC GTC GAT ACA TGC AAG CGT TTA CGT CTC ATG 627
codon-optimized GTT GGA GAT GTT CCC TGG CCA ATG TTC GTG GAT ACA TGT AAG AGA CTG AGA CTG ATG 627
              wt AAA GGA TCG GAT GCC ATT GGT CTC GCT CCG AGG GCG ATG GAG AAG TGC AAG AGC AGA 684
codon-optimized AAG GGA TCT GAC GCC ATT GGA CTG GCT CCA AGA GCA ATG GAA AAG TGC AAG TCA AGG 684
              wt GCT 687
codon-optimized GCT 687
```

Supplementary Figure 4

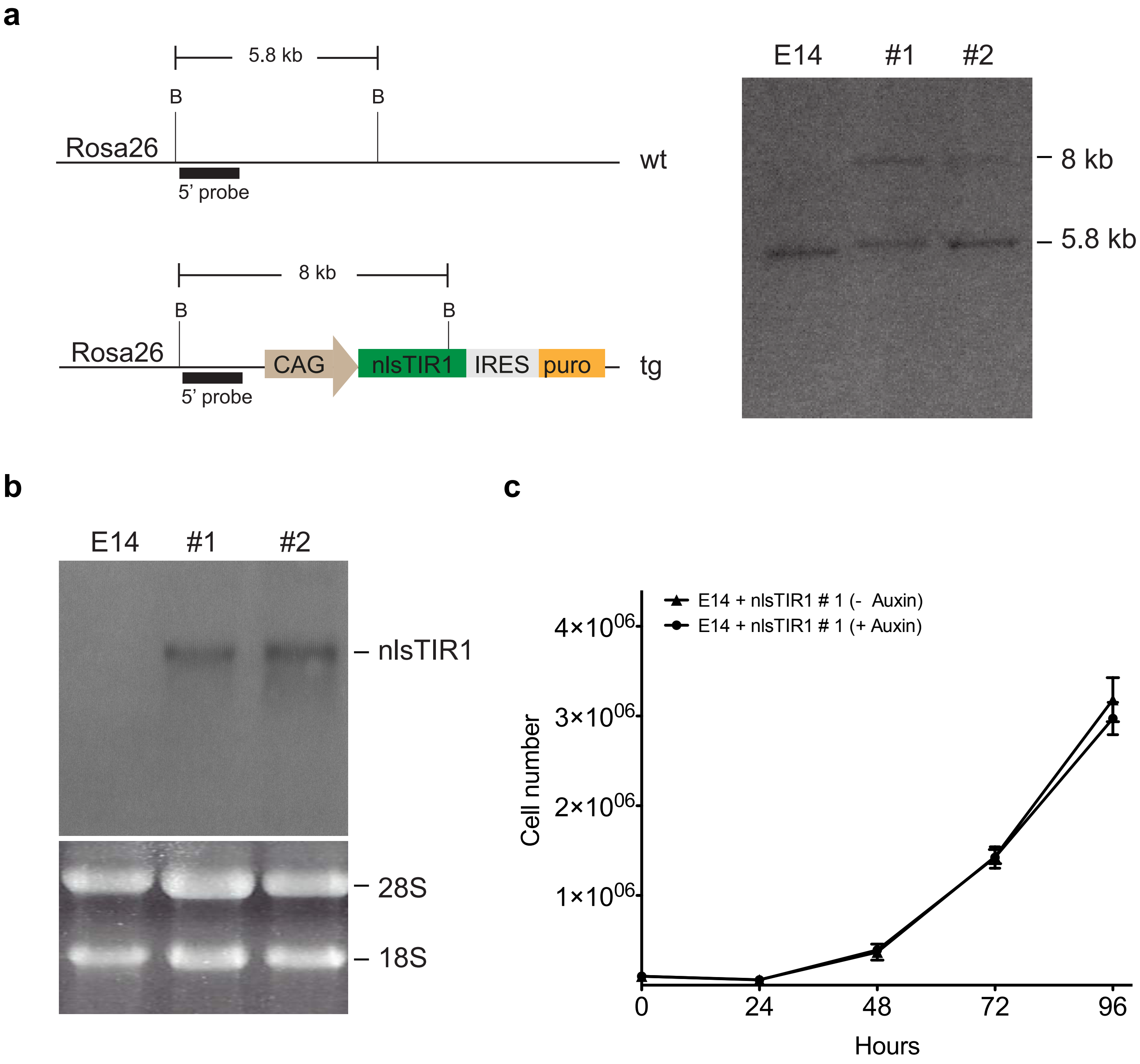

Supplementary Figure 5

Ash2l-locus

TGG GAA CCC TAA CCA GTC CTT GCT TCT GGT GAC ACT GTA AAA TAA TCC TGG   wt
gRNA-1

TGG GAA CCC TAA CCA GTC CTT GCT TCT GGT GAC ACT GTA AAA TAA TCC TGG   wt / 4x

TGG GAA CCC TAA CCA GTC - - - GCT TCT GGT GAC ACT GTA AAA TAA TCC TGG   - 3 bp / 4x

TGG GAA CCC TAA CCA G - - - - - - - - TCT GGT GAC ACT GTA AAA TAA TCC TGG   - 8 bp / 3x

TGG GAA CCC TAA CCA GTC - - - - - - TCT GGT GAC ACT GTA AAA TAA TCC TGG   - 6 bp

TGG GAA CCC TAA CCA GTC - - - - CT TCT GGT GAC ACT GTA AAA TAA TCC TGG   - 4 bp

TGG GAA CCC TAA CCA GTC CTT GCT TCT GGT GAC ACT GTA AAA TAA TCC TGG   + 9 bp
C TG GTT ACA

Supplementary Figure 6

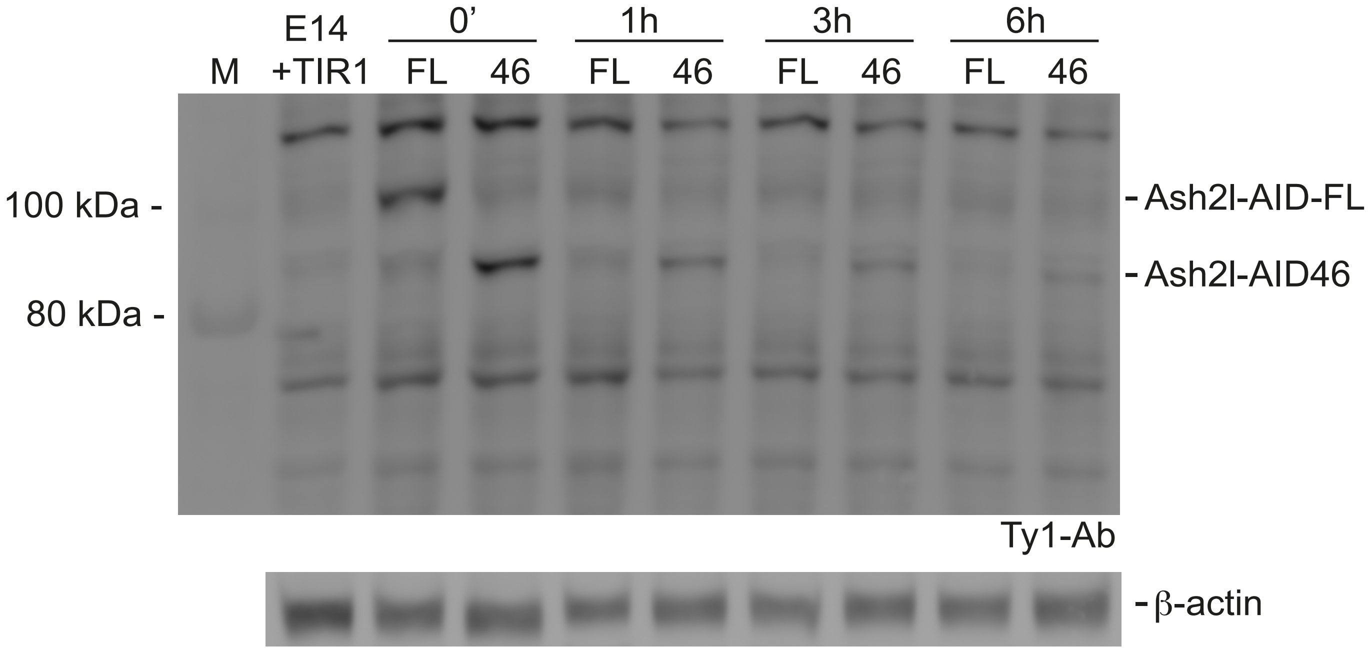

Supplementary Table 1

Name	Sequence 5' to 3'
**ssOligo**	Homology arms underlined
50mer	AAGGACGAAACACCGGTCTCCCATGCATTCAAACTGGTTTAAGAGCTATG
60mer	GTGGAAAGGACGAAACACCGGTCTCCCATGCATTCAAACTGGTTTAAGAGCTATGCTGGA
84mer	CTTTATATATCTTGTGGAAAGGACGAAACACCGGTCTCCCATGCATTCAAACTGGTTTAAGAGCTATGCTGGAAACAGCATAGC
**4-way cloning**	The colours correspond to those used in Figure 2a
Ash2l-AID46-1-fwd	TGCTTAATGAATTACAACAGTACTGCGATGAGTGGCAGGGCGGGGCGTAACATATGCTCGAGGGGAGATCAGGGGTCGGCAT
Ash2l-AID46-1-rev	TCTTGGTTTGTGTGGACTTCATCCAGCGGGTCCTGATTGGTATGCACTTCGGGTTCCCAGGGTGGACTAC
Ash2l-AID46-2-fwd	ATGGAGACTATAAAGATCATGACATTGACTACAAGGATGACGACGACAAGTGAATAATCCTGGGGGTTTTGTT
Ash2l-AID46-2-rev	TATCTCTTCAAATGTAGCACCTGAAGTCAGCCCCATACGATATAAGTTGTCTCGAGCATATGTACTTAGAATATAATTCTGC
Wdr82-AID46-1-fwd	TGCTTAATGAATTACAACAGTACTGCGATGAGTGGCAGGGCGGGGCGTAACTCGAGCATATGGGAGTTTAGTCTAGCCTTGGT
Wdr82-AID46-1-rev	TCTTGGTTTGTGTGGACTTCATCCAGCGGGTCCTGATTGGTATGCACTTCGTCATCAATGGTGGGCAACC
Wdr82-AID46-2-fwd	ATGGAGACTATAAAGATCATGACATTGACTACAAGGATGACGACGACAAGTGAGGAAAAGCCAGAGGTGACTG
Wdr82-AID46-2-rev	TATCTCTTCAAATGTAGCACCTGAAGTCAGCCCCATACGATATAAGTTGTCTCGAGCATATGGGTCATATATAAAAAGGCTAA
**5-way cloning**	The colours correspond to those used in Suppl. Figure 2
PCR-1-fwd	TGCTTAATGAATTACAACAGTACTGCGATGAGTGGCAGGGCGGGGCGTAATACGTACAGAAAACACTTCCCAGAAGATGTCA
PCR-1-rev	TTCCGGCGCCGGATGAGCTGACACTACGCAGAAACATGCTCGAAACACGCGGGCACATGACCCAGGGTGTACATGT
PCR-2-fwd	TAGAAAGCTTCTCTGTTCGTCGTCGTCTACACCTACAGGATGATGGACACAAATATGCACCATGGGTACACATG
PCR-2-rev	GCAATTATTCAGAGCTAAATATCAAAGTAATTCCAACCAGCTCTTAGGCATAACTTCGTATAATGTATGCTATACGAAGTTATCCTAAGTGTGCTAATCCAGATCTCT
PCR-3-fwd	GCCTAAGAGCTGGTTGGAATTACTTTGA
PCR-3-rev	TATCTCTTCAAATGTAGCACCTGAAGTCAGCCCCATACGATATAAGTTGTTACGTACTGCTAGAAGTTCTACTGGATTCTTC
**Insertion of rpsl-genta into BAC**	The colours correspond to those used in Figure 2c
Ash2l-AID-U5	ATCACGTGGAGACAGAAGTGGACGGAAGACGTAGTCCACCCTGGGAACCCGAAGTGCATACCAATCAGGACCCGC
Ash2l-AID-D3	GGAAAACATTTGACACTTTATAAAAACAAAAACAAAACCCCCAGGATTATTCACTTGTCGTCGTCATCCTTGTAGT
Wdr82-AID-U5	GCTGATTTGTGTTTTCCTTCCAGGCCTTCTGGTTGCCCACCATTGATGACGAAGTGCATACCAATCAGGACCCGC
Wdr82-AID-D3	TCAAATGATCAATCAATCTCACTGTCATCTCAGTCACCTCTGGCTTTTCCTCACTTGTCGTCGTCATCCTTGTAGTC
**Subcloning from BAC**	
Ash2-AID-G3	TCTTGGAATCTCAAATGTGATCTTCCTATTTCTTCCCTAAAGTACTGAAGTTTATGACACTGCACCTAGCGATCGCACCGGTGACCCGGGTCTTAATTAATAAGATGATCTTCTTGAGATCG
Ash2-AID-G5	AAAACAAAACGGGATAGAGGGGCTAGAAAGAACTCAGCTGTTACAGGTACTGGCTGTCCTTCCAGAGAACGTCGACGCCGGCGACTTAAGTCTTACCAATGCTTAATCAGTGAGG
Wdr82-AID-G5	ACTGCTCTGTCCTGTCTTCCCTGAATAACAAGTAGAAAATGAAATCTAAGATGAGGAAATAGGTGTCTTGACGTCACCGGTGACCCGGGTCTTAATTAATAAGATGATCTTCTTGAGATCG
Wdr82-AID-G3	TGTATCAAGATAAATAGGAGTTGAAGCAAAATATGAGCAAAAATTGGATGTGAACCCTAAACCACTCTGCGATCGCCGGCGACTTAAGTCTTACCAATGCTTAATCAGTGAGG
**gRNAs**	target sequence underlined
Ash2l-gRNA-1	CGATTTCTTGGCTTTATATATCTTGTGGAAAGGACGAAACACCGTGTCACCAGAAGCAAGGACGTTTTAGAGCTAGAAATAGCAAGTTAAAATAAGGCTAGTCCG
Ash2l-gRNA-2	CGATTTCTTGGCTTTATATATCTTGTGGAAAGGACGAAACACCGTGACACTGTAAAATAATCCGTTTTAGAGCTAGAAATAGCAAGTTAAAATAAGGCTAGTCCG
Wdr82-gRNA-1	CGATTTCTTGGCTTTATATATCTTGTGGAAAGGACGAAACACCGCTGGGCTATTTATACAGTGGTTTTAGAGCTAGAAATAGCAAGTTAAAATAAGGCTAGTCCG
**Southern probes**	
Ash2l-AID-3-fwd	GATGGATCCACACATGAGCACCTACATTCAC
Ash2l-AID-3-rev	CAGTACAAGCTTGTCATAATCTTGATCCCCACTTC
Wdr82-AID-3-fwd	GATCGAATTCAAAAGCTGAAAGATCCCAAG
Wdr82-AID-3-rev	GATGGATCCTTTGTTGAGTAAAATGGGCAAG
Rosa 5-fwd	AGGCGCCCGATAGAATAAATT
Rosa 5-rev	TTTGCCTGGGTATTGCCTAC
**Genotyping PCR**	
Ash2l-AID46-fwd	TACAAGAGCTGCACGGTACG
Ash2l-AID46-rev	GAGCCATCATGTGGTTGTTG
Wdr82-AID46-fwd	CTTGATTCTCCTGGCTCTGC
Wdr82-AID46-rev	AAGCTCTGCTTCCCGTTGTA
BGH-Ash2l-fwd	GGTTAGAAGGCACAGTCGAGGGAATTCGTAAGGACGCCTGCATTCAT
Ash2l-rev	ACCAAGCTTCTCGCTCTTTGGAACACACA
